# *Spirulina* in Clinical Practice: Evidence-Based Human Applications

**DOI:** 10.1093/ecam/nen058

**Published:** 2010-10-19

**Authors:** P. D. Karkos, S. C. Leong, C. D. Karkos, N. Sivaji, D. A. Assimakopoulos

**Affiliations:** ^1^Department of Otolaryngology, Liverpool University Hospitals, Liverpool, UK; ^2^Department of Surgery, Hippocrateio Hospital, Thessaloniki, Greece; ^3^Department of Otolaryngology, University of Ioannina, Ioannina, Greece

## Abstract

*Spirulina* or *Arthrospira* is a blue-green alga that became famous after it was successfully used by NASA as a dietary supplement for astronauts on space missions. It has the ability to modulate immune functions and exhibits anti-inflammatory properties by inhibiting the release of histamine by mast cells. Multiple studies investigating the efficacy and the potential clinical applications of *Spirulina* in treating several diseases have been performed and a few randomized controlled trials and systematic reviews suggest that this alga may improve several symptoms and may even have an anticancer, antiviral and antiallergic effects. Current and potential clinical applications, issues of safety, indications, side-effects and levels of evidence are addressed in this review. Areas of ongoing and future research are also discussed.

## 1. Introduction

*Spirulina* is a microscopic and filamentous cyanobacterium that derives its name from the spiral or helical nature of its filaments. It has a long history of use as food and it has been reported that it has been used during the Aztec civilization [1]. *Spirulina* refers to the dried biomass of *Arthrospira platensis*, an oxygenic photosynthetic bacterium found worldwide in fresh and marine waters. This alga represents an important staple diet in humans and has been used as a source of protein and vitamin supplement in humans without any significant side-effects. Apart from the high (up to 70%) content of protein, it also contains vitamins, especially B_12_ and provitamin A (*β*-carotenes), and minerals, especially iron. It is also rich in phenolic acids, tocopherols and *γ*-linolenic acid [1]. *Spirulina* lacks cellulose cell walls and therefore it can be easily digested [1].

Many toxicological studies have proven *Spirulina*'*s* safety. *Spirulina* now belongs to the substances that are listed by the US Food and Drug Administration under the category Generally Recognized as Safe (GRAS) [2–6]. *Spirulina* is relatively easy to cultivate but flourishes only in alkaline lakes with an extremely high pH and in large outdoor ponds under controlled conditions. There are only a few areas worldwide that have the ideal sunny climate for production of this alga, including Greece (Nigrita, Serres), Japan, India, United States and Spain. Currently, *Spirulina* can be found in health food stores and is sold mainly as a dietary supplement in the form of health drinks or tablets. Microalgae have been used for more than 10 years as dietary supplements without significant side-effects [7]. The aims of this review are to summarize the mechanisms of action, highlight the potential effects of this alga in humans and address current and possible future clinical applications, based mainly on *in vivo* studies and a few well-designed *in vitro* studies and the highest levels of evidence available in the literature.

## 2. Evidence-Based Applications of *Spirulina*

### 2.1. *Spirulina* and Chronic Fatigue

*Spirulina* has been promoted as “the food of the future" with “exceptional constituents" that contribute to high energy levels. A few of these constituents such as polysaccharides (Rhamnose and Glycogen) and essential fat (GLA) are absorbed easily by human cells and help in energy release. *Spirulina* increases healthy lactobacillus in the intestine, enabling the production of Vitamin B6 that also helps in energy release. Despite this promotion, the only available placebo-controlled randomized trial showed that the scores of fatigue were not significantly different between *spirulina* and placebo. Spirulina administered at a dose of 3 g day^−1^ did not ameliorate fatigue more than the placebo in any of the four subjects and possibly it has no effect on chronic fatigue [8].

### 2.2. Allergy, Rhinitis, and Immunomodulation

It has been well documented that *Spirulina* exhibits anti-inflammatory properties by inhibiting the release of histamine from mast cells [9, 10].

In a recent randomized, double-blind placebo-controlled trial [11], individuals with allergic rhinitis were fed daily, either with placebo or *Spirulina* for 12 weeks. Peripheral blood mononuclear cells were isolated before and after the *Spirulina* feeding and levels of cytokines (interleukin-4 (IL-4), interferon-*γ* (IFN-*γ*) and interleukin-2), which are important in regulating immunoglobulin (Ig)E-mediated allergy, were measured. The study showed that high dose of *Spirulina* significantly reduced IL-4 levels by 32%, demonstrating the protective effects of this microalga toward allergic rhinitis.

Ishii et al. [12] studied the influence of *Spirulina* on IgA levels in human saliva and demonstrated that it enhances IgA production, suggesting a pivotal role of microalga in mucosal immunity.

A Japanese team identified the molecular mechanism of the human immune capacity of *Spirulina* by analysing blood cells of volunteers with pre- and post-oral administration of hot water extract of *Spirulina platensis*. IFN-*γ* production and Natural Killer (NK) cell damage were increased after administration of the microalga extracts to male volunteers [13].

In a recent double-blind, placebo-controlled study from Turkey evaluating the effectiveness and tolerability of *Spirulina* for treating patients with allergic rhinitis, *Spirulina* consumption significantly improved the symptoms and physical findings compared with placebo (*P* < .001), including nasal discharge, sneezing, nasal congestion and itching [14].

It is well understood that deficiency of nutrients is responsible for changes in immunity, which manifests as changes in production of T-cells, secretory IgA antibody response, cytokines and NK-cell activity. The above studies suggest that *Spirulina* may modulate the immune system by its role in covering nutritional deficiencies.

### 2.3. Antiviral Applications: *In Vitro* Studies

There are no *in vivo* studies providing strong evidence supporting the possible antiviral properties of *Spirulina*. The active component of the water extract of *S. platensis* is a sulfated polysaccharide, calcium spirulan (Ca-Sp). According to Hayashi et al. [15], Ca-Sp inhibits the *in vitro* replication of several enveloped viruses including Herpes simplex type I, human cytomegalovirus, measles and mumps virus, influenza A virus and human immunodeficiency virus-1 virus (HIV-1).

Another more recent study showed *in vitro* that an aqueous extract of *S. platensis* inhibited HIV-1 replication in human T-cells, peripheral blood mononuclear cells and Langerhan cells [16]. The advantage of using herbs and algal products with proven antiviral properties in fighting certain viruses is that they can be used—through immunomodulation—even when the infection is established.

Of course, the above promising effects need to be studied further in animal models and humans before any definitive conclusions are drawn.

### 2.4. Cholesterol-Lowering Effects and Effects on Diabetes

Cardiovascular disease remains the number one cause of death in developed countries, despite increased awareness, and high cholesterol is one of the most important risk factors in atherosclerosis.

Nakaya et al. [17], in the first human study, gave 4.2 g day^−1^ of *Spirulina* to 15 male volunteers and, although there was no significant increase in high-density lipoprotein (HDL) levels, they observed a significant reduction of high-density lipoprotein (LDL) cholesterol after 8 weeks of treatment. The atherogenic effect also declined significantly in the above group [17].

Ramamoorthy and Premakumari [18] in a more recent study administered *Spirulina* supplements in ischemic heart disease patients and found a significant reduction in blood cholesterol, triglycerides and LDL cholesterol and an increase in HDL cholesterol. More research is needed before *Spirulina* can be recommended to lower cholesterol levels but its role as a natural food supplement in combating hyperlipidaemia, in combination with other therapeutic options, should not be overlooked.

Finally, Mani et al. [19] in a clinical study, found a significant reduction in LDL : HDL ratio in 15 diabetic patients who were given *Spirulina*. However, this study was small and better studies are needed before *Spirulina* can be recommended in diabetes.

### 2.5. Anticancer Effects

It has been argued that the combined antioxidant and immune modulation characteristics of *Spirulina* may have a possible mechanism of tumor destruction and hence play a role in cancer prevention. Whilst there are many animal and *in vitro* studies, there has been only one trial with human subjects. This study looked specifically at the effects of *Spirulina* on oral carcinogenesis, in particular leukoplakia [20]. It is not surprising that few human studies exist to date as cancer prevention trials with lower cancer incidence as an endpoint have logistic problems, rendering them essentially impossible to conduct for most malignancies. The study conducted by Mathew et al. on a cohort of 77 patients originates from previous trials on hamsters that showed tumor regression after topical application or enteral intake of *Spirulina* extract [21–23]. They reported that 45% of their study cohort showed complete regression of leukoplakia after taking *Spirulina* supplements for 1 year. The authors also reported that there was no rise in the serum concentration of retinal *β*-carotene despite supplementation and concluded that other constituents within *Spirulina* may have been responsible for the anticancer effects. Whilst their results appear promising, it was an unblinded, non-randomized trial and as such cannot be regarded as evidence of a positive effect.

### 2.6. Chronic Arsenic Poisoning: A Randomized Trial

Millions of people in Bangladesh, India, Taiwan and Chile are consuming high concentration of arsenic through drinking water and are at risk of chronic arsenic poisoning for which there is no specific treatment. A placebo-controlled, double-blind study was conducted to evaluate the effectiveness of spirulina extract plus zinc in the treatment of chronic arsenic poisoning [24]. Forty-one patients with chronic arsenic poisoning were randomly treated by either placebo (17 patients) or spirulina extract (250 mg) plus zinc (2 mg) (24 patients) twice daily for 16 weeks. Each patient was supplied with arsenic-safe drinking water by installing a locally made water filter at household level. Effectiveness of spirulina extract plus zinc was evaluated by comparing changes in skin manifestations (clinical scores) and arsenic contents in urine and hair, between the placebo- and spirulina extract plus zinc-treated groups. Results showed that spirulina extract plus zinc twice daily for 16 weeks may be useful for the treatment of chronic arsenic poisoning with melanosis and keratosis. More randomized trials are required but the results are promising.

### 2.7. Antioxidant Effects: No *In Vivo* Studies

C-phycocyanin (C-PC) is one of the major biliproteins of *Spirulina* with antioxidant and radical scavenging properties. C-PC, a selective cyclooxygenase-2 inhibitor, induces apoptosis in lipopolysaccharide-stimulated RAW 264.7 macrophages. It is also known to exhibit anti-inflammatory and anticancer properties [25]. To date though, there are no *in vivo* human studies on possible antioxidant effects of *Spirulina*.

## 3. Conclusions

The positive effects of *Spirulina* in allergic rhinitis are based on adequate evidence but larger trials are required. It is believed that the anticancer effects of *Spirulina* are perhaps derived from *β*-carotene, a known antioxidant; however, the link between *β*-carotene level and carcinogenesis cannot be established as the etiology of carcinoma is frequently multifactorial [26, 27]. There are some positive studies on the cholesterol-lowering effects of *Spirulina* but larger studies are required before any definitive conclusions can be made. Finally, there are no high-level evidence trials on the role played by *Spirulina* in chronic fatigue and in antiviral applications. At the moment, what the literature suggests is that *Spirulina* is a safe food supplement without significant side-effects but its role as a drug remains to be seen.

## References

[1] Dillon JC, Phuc AP, Dubacq JP (1995). Nutritional value of the alga Spirulina. *World Review of Nutrition and Dietetics*.

[2] Tarantino LM Agency Response Letter GRAS Notice No. GRN000127.

[3] Salazar M, Chamorro G, Salazar S, Steele C (1996). Effect of *Spirulina maxima* consumption on reproductive and peri- and postnatal development in rats. *Food and Chemical Toxicology*.

[4] Chamorro G, Salazar S, Favila-Castillo L, Steele C, Salazar M (1997). Reproductive and peri-and postnatal evaluation of *Spirulina maxima* in mice. *Journal of Applied Phycology*.

[5] Salazar M, Martínez E, Madrigal E, Ruiz LE, Chamorro GA (1998). Subchronic toxicity study in mice fed *Spirulina*. *Journal of Ethnopharmacology*.

[6] Belay A (2002). The potential application of *Spirulina (Arthrospira)* as a nutritional and therapeutic supplement in Health management. *Journal of the American Nutraceutical Association*.

[7] Kay RA (1991). Microalgae as food and supplement. *Critical Reviews in Food Science and Nutrition*.

[8] Baicus C, Baicus A (2007). Spirulina did not ameliorate idiopathic chronic fatigue in four N-of-1 randomized controlled trials. *Phytotherapy Research*.

[9] Yang H-N, Lee E-H, Kim H-M (1997). *Spirulina platensis* inhibits anaphaylactic reaction. *Life Sciences*.

[10] Kim H-M, Lee E-H, Cho H-H, Moon Y-H (1998). Inhibitory effect of mast cell-mediated immediate-type allergic reactions in rats by *Spirulina*. *Biochemical Pharmacology*.

[11] Mao TK, van de Water J, Gershwin ME (2005). Effects of a Spirulina-based dietary supplement on cytokine production from allergic rhinitis patients. *Journal of Medicinal Food*.

[12] Ishii K, Katoch T, Okuwaki Y, Hayashi O (1999). Influence of dietary *Spirulina* platensis on IgA level in human saliva. *Journal of Kagawa Nutrition University*.

[13] Hirahashi T, Matsumoto M, Hazeki K, Saeki Y, Ui M, Seya T (2002). Activation of the human innate immune system by Spirulina: augmentation of interferon production and NK cytotoxicity by oral administration of hot water extract of *Spirulina platensis*. *International Immunopharmacology*.

[14] Cingi C, Conk-Dalay M, Cakli H, Bal C The effects of spirulina on allergic rhinitis.

[15] Hayashi K, Hayashi T, Maedaa M, Kojima I (1996). Calcium spirulan, an inhibitor of envelope virus replication, from a blue-green alga *Spirulina platensis*. *Journal of Natural Products*.

[16] Ayehunie S, Belay A, Baba TW, Ruprecht RM (1998). Inhibition of HIV-1 replication by an aqueous extract of *Spirulina platensis (Arthrospira platensis)*. *Journal of Acquired Immune Deficiency Syndromes and Human Retrovirology*.

[17] Nakaya N, Homa Y, Goto Y (1988). Cholesterol lowering effect of *Spirulina*. *Atherosclerosis*.

[18] Ramamoorthy A, Premakumari S (1996). Effect of supplementation of *Spirulina* on hypercholesterolemic patients. *Journal of Food Science and Technology*.

[19] Mani UV, Desai S, Iyer U (2000). Studies on the long-term effect of *Spirulina* supplementation on serum lipid profile and glycated proteins in NIDDM patients. *Journal of Nutraceuticals, Functional and Medical Foods*.

[20] Mathew B, Sankaranarayanan R, Nair PP (1995). Evaluation of chemoprevention of oral cancer with Spirulina fusiformis. *Nutrition and Cancer*.

[21] Shklar G, Schwartz J (1988). Tumor necrosis factor in experimental cancer regression with alphatocopherol, beta-carotene, canthaxanthin and algae extract. *European Journal of Cancer and Clinical Oncology*.

[22] Schwartz J, Shklar G, Reid S, Trickler D (1988). Prevention of experimental oral cancer by extracts of Spirulina-Dunaliella algae. *Nutrition and Cancer*.

[23] Schwartz J, Shklar G (1987). Regression of experimental hamster cancer by beta carotene and algae extracts. *Journal of Oral and Maxillofacial Surgery*.

[24] Misbahuddin M, Islam AZ, Khandker S, Al-Mahmud I, Islam N, Anjumanara (2006). Efficacy of spirulina extract plus zinc in patients of chronic arsenic poisoning: a randomized placebo-controlled study. *Clinical Toxicology*.

[25] Reddy MC, Subhashini J, Mahipal SVK (2003). C-Phycocyanin, a selective cyclooxygenase-2 inhibitor, induces apoptosis in lipopolysaccharide-stimulated RAW 264.7 macrophages. *Biochemical and Biophysical Research Communications*.

[26] Malila N, Virtamo J, Virtanen M, Pietinen P, Albanes D, Teppo L (2002). Dietary and serum alpha-tocopherol, beta-carotene and retinol, and risk for colorectal cancer in male smokers. *European Journal of Clinical Nutrition*.

[27] Liede K, Hietanen J, Saxen L, Haukka J, Timonen T, Häyrinen-Immonen R (1998). Long-term supplementation with alpha-tocopherol and beta-carotene and prevalence of oral mucosal lesions in smokers. *Oral Diseases*.

